# HPLC Analysis of tRNA‐Derived Nucleosides

**DOI:** 10.21769/BioProtoc.5213

**Published:** 2025-02-20

**Authors:** Xingxing Chen, Fu Xu

**Affiliations:** 1 Department of Radiation Oncology, Fudan University Shanghai Cancer Center, Shanghai, China; 2 Department of Oncology, Shanghai Medical College, Fudan University, Shanghai, China; 3 Division of Mechanisms Regulating Gene Expression, German Cancer Research Center (DKFZ), Heidelberg, Germany

**Keywords:** tRNA modifications, HPLC, Retention time, UV absorbance, Epitranscriptome, Yeast

## Abstract

Transfer RNAs (tRNAs), the essential adapter molecules in protein translation, undergo various post-transcriptional modifications. These modifications play critical roles in regulating tRNA folding, stability, and codon–anticodon interactions, depending on the modified position. Methods for detecting modified nucleosides in tRNAs include isotopic labeling combined with chromatography, antibody-based techniques, mass spectrometry, and high-throughput sequencing. Among these, high-performance liquid chromatography (HPLC) has been a cornerstone technique for analyzing modified nucleosides for decades. In this protocol, we provide a detailed, streamlined approach to purify and digest tRNAs from yeast cells and analyze the resulting nucleosides using HPLC. By assessing UV absorbance spectra and retention times, modified nucleosides can be reliably quantified with high accuracy. This method offers a simple, fast, and accessible alternative for studying tRNA modifications, especially when advanced technologies are unavailable.

Key features

• A streamlined protocol for purifying total tRNAs from yeast cells.

• Adaptable for other RNA species and organisms, provided sufficient input material.

• Enables the quantification of approximately 20 types of tRNA modifications.

• Offers a cost-effective and rapid alternative for analyzing tRNA modifications by HPLC method.

## Background

The landscape of RNA modifications across the transcriptome, known as the epitranscriptome, is receiving increasing attention. To date, over 170 distinct types of RNA modifications have been identified [1]. These modifications play pivotal roles in regulating all aspects of RNA biology, from transcription and processing to degradation, ultimately influencing cell fate. Among all RNA species, tRNA stands out as one of the most extensively modified, with an average of 13 modifications per molecule [2]. These modifications encompass a wide range of chemical alterations, from simple methylations to complex structures like pseudouridylation. Deficiencies in tRNA modifications or the enzymes responsible for them have been linked to various human diseases, including neurological disorders and cancer [3]. Thus, studying tRNA modifications is critical for advancing our understanding of human health.

Various methods are available for the quantitative analysis of tRNA modifications. Among these, mass spectrometry, regarded as the gold standard for tRNA modification identification, is widely used in the field. Additionally, emerging high-throughput sequencing technologies, such as mim-tRNAseq and nano-tRNAseq, are revolutionizing the study of tRNA modifications by enabling single-nucleoside resolution analysis at the epitranscriptome level [4,5]. Among the classic methods, for decades HPLC has been a cornerstone technique for analyzing modified nucleosides, remaining a valuable tool in RNA modification research. Here, we present a modified version of the protocol originally described by Gehrke et al. [6]. This updated protocol employs a C30 reverse-phase column coated with a 30-carbon-long alkyl chain, offering a more hydrophobic separation phase compared to the previously used C18 column, which features an 18-carbon-long alkyl chain. Additionally, we provide a streamlined protocol for purifying tRNAs from yeast cells using diethylaminoethyl cellulose (DE52) for subsequent HPLC analysis. The cellulose matrix exhibits weak ion exchange properties, enabling the separation of RNAs of different sizes under varying salt concentrations [7]. With this approach, we can reliably separate and identify more than 20 types of modified nucleosides with high accuracy and confidence.

## Materials and reagents

**Biological materials**

1. *Saccharomyces cerevisiae*

**Reagents**

1. Synthetic complete yeast growth medium (Formedium, catalog number: CSC0201)

2. Nuclease-free water (Invitrogen, catalog number: 10977-035)

3. NaCl (Sigma-Aldrich, catalog number: S9888)

4. Water-saturated phenol (AppliChem, catalog number: A1624)

5. Chloroform (Sigma-Aldrich, catalog number: 650498)

6. Ethanol (Sigma-Aldrich, catalog number: 1009861000)

7. 1 M Tris-HCl, pH 7.4 (Santa Cruz, catalog number: SC-301950)

8. 1 M Tris-HCl, pH 8.3 (Fisher Scientific, catalog number: T1083)

9. Qubit RNA Broad Range kit (Thermo Fisher, catalog number: Q10210)

10. Isopropanol (Sigma-Aldrich, catalog number: 1096341011)

11. DMSO (Sigma-Aldrich, catalog number: 34869)

12. Diethylaminoethyl cellulose (DE52) (Biophoretics, catalog number: B45059)

13. Nuclease P1 (Sigma-Aldrich, catalog number: N8630)

14. Bacterial alkaline phosphatase (BAP) (Sigma-Aldrich, catalog number: P4252)

15. ZnCl_2_ (Sigma-Aldrich, catalog number: 39059)

16. 1 M sodium acetate, pH 5.0 (Thermo Fisher, catalog number: J60964.AK)

17. NH_4_H_2_PO_4_ solution (Sigma-Aldrich, catalog number: 216003)

18. NH_4_OH solution (Sigma-Aldrich, catalog number: 221228)

19. H_3_PO_4_ solution (Sigma-Aldrich, catalog number: 695017)

20. Methanol (Honeywell Chemicals, catalog number: 34966)

21. Acetonitrile (Honeywell Chemicals, catalog number: 34881)

**Solutions**

1. 0.9% NaCl (w/v)

2. 10 mM ZnCl_2_

3. DE52 binding buffer (see Recipes)

4. DE52 solution (see Recipes)

5. tRNA elution buffer (see Recipes)

6. Nuclease P1 solution (see Recipes)

7. HPLC solvent A (see Recipes)

8. HPLC solvent B (see Recipes)

9. HPLC solvent C (see Recipes)

**Recipes**

**1. 0.9% NaCl**

Store at RT.

**Table:** 

Reagent	Final concentration	Amount
NaCl	0.9% (w/v)	9 g
Nuclease-free H_2_O	n/a	1,000 mL

**2. 10 mM ZnCl_2_**

Store at -20 °C. Stable for at least five years.

**Table:** 

Reagent	Final concentration	Amount
0.1 M ZnCl_2_	0.01 M	100 μL
Nuclease-free H_2_O	n/a	900 μL

**3. DE52 binding buffer**

Store at RT. Stable for at least one year.

**Table:** 

Reagent	Final concentration	Amount
1 M Tris-HCl, pH 7.4	0.1 M	100 mL
NaCl	0.1 M	5.844 g
Nuclease-free H_2_O	n/a	900 mL

**4. DE52 solution**

Store at 4 °C. Stable for at least six months.

**Table:** 

Reagent	Final concentration	Amount
DE52	0.33 g/mL (w/v)	100 g
DE52 binding buffer	n/a	300 mL

**5. tRNA elution buffer**

Store at RT. Stable for at least one year.

**Table:** 

Reagent	Final concentration	Amount
1 M Tris-HCl, pH 7.4	0.1 M	100 mL
NaCl	1 M	58.44 g
Nuclease-free H_2_O	n/a	900 mL

**6. Nuclease P1 solution**

Store at -20 °C. Stable for at least one year.

**Table:** 

Reagent	Final concentration	Amount
Nuclease P1	>250 units /1 mL	1 vial (>250 units)
1 M sodium acetate, pH 5.0	30 mM	30 μL
Nuclease-free H_2_O	n/a	970 μL

**7. HPLC solvent A**

Prepare before use. Adjust pH to 5.3 with NH_4_OH and H_3_PO_4_.

**Table:** 

Reagent	Final concentration	Amount
1 M NH_4_H_2_PO_4_	0.01 M	10 mL
Methanol	2.5% (v/v)	25 mL
Milli-Q H_2_O	n/a	965 mL

**8. HPLC solvent B**

Prepare before use. Adjust pH to 5.1 with NH_4_OH and H_3_PO_4_.

**Table:** 

Reagent	Final concentration	Amount
1 M NH_4_H_2_PO_4_	0.01 M	10 mL
Methanol	20% (v/v)	200 mL
Milli-Q H_2_O	n/a	790 mL

**9. HPLC solvent C**

Prepare before use.

**Table:** 

Reagent	Final concentration	Amount
1 M NH_4_H_2_PO_4_	0.01 M	10 mL
Acetonitrile	35% (v/v)	350 mL
Milli-Q H_2_O	n/a	640 mL

**Laboratory supplies**

1. Disposable gloves

2. Milli-Q water

3. 15 mL centrifuge tubes (Corning, catalog number: 430052)

4. 50 mL centrifuge tubes (Corning, catalog number: 352070)

5. 1.5 mL micro-centrifuge tubes (Eppendorf, catalog number: 0030120086)

6. 1 L glass bottles (Thermo Fisher, catalog number: FB8001000)

7. HPLC sample tube (Waters, catalog number: 186000273)

8. HPLC sample tube cap (Waters, catalog number: 186000305)

9. HPLC sample insert (Waters, catalog number: WAT094170)

10. Polypropylene column (Bio-Rad, catalog number: 731-1550)

11. Reversed phase aqueous C30 column (Phenomenex, catalog number: CH0-5690)

## Equipment

1. Single channel pipette set (Sigma-Aldrich, catalog number: EP3123000918-1EA)

2. Qubit fluorometer (Thermo Fisher, catalog number: Q33238; or similar)

3. 30 °C shaking incubator (INFORS-HT, model: Ecotron; or similar)

4. Cell density photometer (Implen, model: DiluPhotometer; or similar)

5. Centrifuge for 50 mL conical tube (Heraeus, model: Multifuge X3R; or similar)

6. Benchtop centrifuge for 1.5 mL centrifuge tube (Eppendorf, model: 5427R; or similar)

7. Benchtop mixer (Heidolph Instrument, model: 545-10000-00; or similar)

8. pH meter (Mettler Toledo, model: 30266626; or similar)

9. HPLC separation module (Waters, model: 2695)

10. PDA detector (Waters, model: 2996)

## Software and datasets

1. Empower 3 FR4 (Waters, Version 3 Feature Release 4, released in 2017); requires a license

## Procedure

**A. tRNA isolation**

1. Harvest 100 units (100 mL of yeast culture at OD_600 _= 1) of yeast cells grown in synthetic complete yeast growth medium at log phase in 50 mL centrifuge tubes. Centrifuge the tubes at 1,000× *g* for 3 min at room temperature (RT; 25 °C) and remove the supernatant.

2. Wash the cells once with 3 mL of 0.9% NaCl. Centrifuge at 1,000× *g* at RT for 3 min and remove the supernatant.

3. Resuspend the cells in 8 mL of phenol and vortex at 1,200 rpm using a benchtop mixer for 30 min at RT.

4. Add 400 μL of chloroform and vortex at 1,200 rpm for an additional 15 min at RT. Centrifuge at 12,000× *g* for 20 min at 4 °C.

5. Collect the water phase in a 50 mL conical tube, add 4 mL of water-saturated phenol, and vortex at 1,200 rpm for 15 min at RT.

6. Centrifuge at 12,000× *g* for 20 min at 4 °C. Collect the water phase and mix it with 2.5 volumes of ethanol by inverting the tubes three times.

7. Precipitate at -80 °C for 3 h or -20 °C overnight.

8. Centrifuge at 12,000× *g* for 20 min at 4 °C. Remove the supernatant and dissolve the RNA pellet in 5 mL of DE52 binding buffer.

9. Prepare a DE52 cellulose column by adding 5 mL of thoroughly mixed DE52 solution to a polypropylene column. Let the liquid pass through the column for approximately 5 min by gravity at RT.

10. Add the resuspended RNA pellet to the DE52 cellulose column and let the liquid pass through the column by gravity at RT.

11. Add 7 mL of DE52 binding buffer to the column and wait 5 min. Repeat the step once more.

12. Elute the RNA by adding 7 mL of tRNA elution buffer and collect the elute in a 15 mL centrifuge tube. Elute the tRNA once more by adding the elute back to the column to increase the yield.

13. Add 5 mL of isopropanol to the elute and mix by inverting the tube three times. Precipitate tRNA at -20 °C for at least 3 h.

14. Centrifuge at 12,000× *g* for 20 min at 4 °C to pellet tRNA. Discard the supernatant and resuspend the pellet in 1 mL of 70% EtOH.

15. Transfer the resuspension to a 1.5 mL centrifuge tube and spin at 16,000× *g* for 5 min at 4 °C to pellet tRNA.

16. Dissolve the tRNA in 50 μL of nuclease-free water. Measure the tRNA concentration with Qubit RNA Broad Range kit. Approximately 200 μg of tRNA can be obtained from 100 units of yeast cells.

17. The tRNA can be stored at -80 °C for up to one week or continued with tRNA digestion.

**B. tRNA digestion**

1. Perform nuclease P1 digestion by preparing the following mix and incubating at 37 °C for 16 h.

50 μg tRNA x μL

10 mM ZnCl_2_ 5 μL

Nuclease P1 solution 10 μL

Nuclease-free H_2_O Add to 50 μL

2. Dephosphorylate the P1-digested tRNA by preparing the following mix and incubating at 37 °C for 2 h. Samples are ready for HPLC analysis.

Digested tRNA 50 μL

0.5 M Tris-HCl pH 8.3 mM 20 μL

BAP 4 μL

**C. HPLC analysis**

1. Prepare 1 L of each HPLC solvent (A, B, and C; see Recipes).

2. Put the solvent tubes (marked A, B, or C) into the corresponding HPLC solvent. Solvent tube D will not be used and can be kept in water.

3. Turn on both the HPLC separation module and the PDA detector.

4. Wet prime the HPLC system with a flow rate of 4 mL/min for 4 min.

5. Turn on the degas function.

6. Open Empower 3 software. Set up the gradient according to Table 1.

**Table 1.** Gradient of the HPLC method.

Step	Time (min)	Flow (mL/min)	%A	%B	%C	%D	Curve
1		1.00	100	0	0	0	
2	12	1.00	100	0	0	0	11
3	20	1.00	90	10	0	0	6
4	25	1.00	75	25	0	0	6
5	32	1.00	40	60	0	0	6
6	36	1.00	38	62	0	0	6
7	45	1.00	0	100	0	0	6
8	80	1.00	0	0	100	0	6
9	95	1.00	100	0	0	0	6
10	175	0.05	34	33	33	0	6

7. Set up the auto-injection volume to 60 μL and the running time to 140 min for each sample.

8. Transfer the digested tRNAs to the 2 mL HPLC sample tube with the sample insert and cap.

9. Update the sample inject table starting with nuclease-free water as the first sample and DMSO as the second to clean the C30 column.

10. Continue running the digested tRNA samples on the HPLC.

## Data analysis

**Figure 1.**
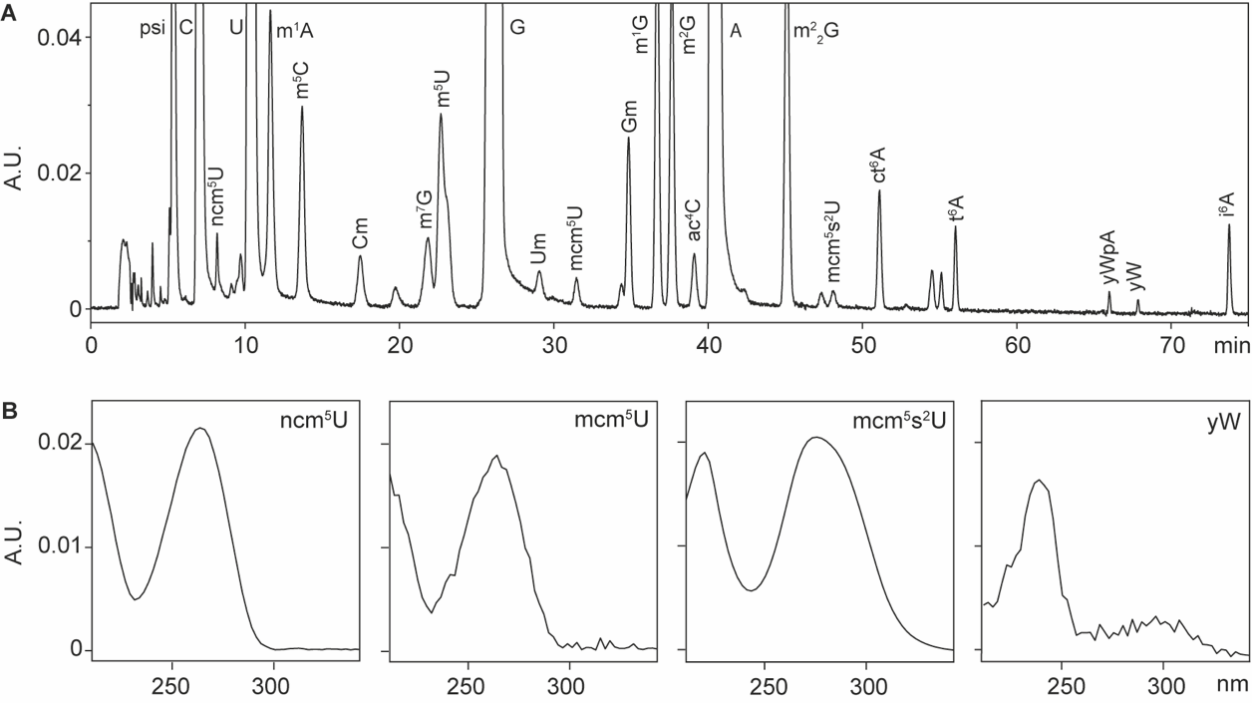
HPLC analysis of tRNA-derived nucleosides. A. Representative HPLC chromatography of modified nucleosides derived from yeast total tRNAs. B. Representative UV absorbance spectra of modified nucleosides. Abbreviations: absorbance unit (A.U.); 5-carbamoylmethyluridine (ncm^5^U); 5-methoxycarbonylmethyluridine (mcm^5^U); 5-methoxycarbonylmethyl-2-thiouridine (mcm^5^s^2^U); wybutosine (yW). Full names of the rest of the abbreviations can be found in [1].

This protocol is adapted from the original HPLC chromatography method [6]. Each nucleoside is characterized by a unique UV absorbance spectrum and retention time, which are used for its identification and quantification. The retention times of the nucleosides are shown in Figure 1A, while the UV absorbance spectra for ncm^5^U, mcm^5^U, mcm^5^s^2^U, and yW are displayed in Figure 1B. Additional UV absorbance spectra can be found in this chapter [6]. For HPLC analysis, we typically include tRNAs isolated from cells deficient in the formation of the target nucleosides as a positive control. For instance, tRNAs from *elp3Δ* cells, which are deficient in producing wobble uridine modifications, are used to analyze ncm^5^U, mcm^5^U, and mcm^5^s^2^U [8].

## Validation of protocol

This protocol has been used and validated in the following research articles:

• Xu et al. [8]. Sod1-deficient cells are impaired in formation of the modified nucleosides mcm^5^s^2^U and yW in tRNA. *RNA* (Figures 1–4, Supplementary Figure S1, and S2).

• Xu et al. [9]. *SSD1* suppresses phenotypes induced by the lack of Elongator-dependent tRNA modifications. *PLOS Genetics* (Supplementary Figure 5).

• Xu et al. [10]. Identification of factors that promote biogenesis of tRNA_CGA_^Ser^. *RNA Biology* (Supplementary Figure S2).

• Xu et al. [11]. Yeast Elongator protein Elp1p does not undergo proteolytic processing in exponentially growing cells. *MicrobiologyOpen* (Figure 4, panel B).

• Chen et al. [12]. Elongator complex influences telomeric gene silencing and DNA damage response by its role in wobble uridine tRNA modification. *PLOS Genetics* (Figure 5).

• Chen et al. [13]. Defects in tRNA modification associated with neurological and developmental dysfunctions in *Caenorhabditis elegans* elongator mutants. *PLOS Genetics* (Figure 2 and supplementary Figure S1–S3).

• Huang et al. [14]. A genome-wide screen identifies genes required for formation of the wobble nucleoside 5-methoxycarbonylmethyl-2-thiouridine in *Saccharomyces cerevisiae. RNA* (Figure 2).

• Björk et al. [15]. A conserved modified wobble nucleoside (mcm^5^s^2^U) in lysyl-tRNA is required for viability in yeast. *RNA* (Figure 5).

• Esberg et al. [16]. Elevated levels of two tRNA species bypass the requirement for elongator complex in transcription and exocytosis. *Molecular Cell* (Supplementary Table 1).

• Huang et al. [17]. An early step in wobble uridine tRNA modification requires the Elongator complex. *RNA* (Table 1 and Figure 3).

## General notes and troubleshooting

1. Various media are available for growing yeast cells, including YEPD and synthetic dropout media with additional supplements. We observed lower background noise during HPLC analysis of tRNA nucleosides from cells grown in synthetic dropout medium.

2. Digested tRNA can be stored at -80 °C and analyzed later. No significant differences were observed in nucleoside analysis for samples stored at -80 °C for up to one week. For longer storage, the stability of modified nucleosides should be evaluated.

3. When a positive control strain is available, it is essential to include tRNAs isolated from the control strain. The retention time of each nucleoside may vary by seconds to minutes, depending on the precision of HPLC solvent preparation.

4. To maintain optimal separation of nucleosides, we typically replace the C30 column after analyzing approximately 50 samples or when the retention times deviate significantly from the expected values.

5. While nucleoside spectra are generally stable, we noticed increased background noise when the UV lamp in the PDA detector has been used for a long period.

6. We typically use 50 μg of tRNA as the starting material for analyzing wobble uridine modifications. Smaller input amounts may suffice for abundant modified nucleosides such as m^1^A, m^5^C, m^1^G, ac^4^C, and t^6^A.

7. The methanol concentration in HPLC solvent A can be increased to 6% (v/v) for the detection of m^3^C [10].

8. This protocol can also be applied to analyze tRNA nucleosides derived from other organisms, provided sufficient input material is available.
